# Finite Element-Based Numerical Simulations to Evaluate the Influence of Wollastonite Microfibers on the Dynamic Compressive Behavior of Cementitious Composites

**DOI:** 10.3390/ma14164435

**Published:** 2021-08-08

**Authors:** Gideon A. Lyngdoh, Sami Doner, Sumeru Nayak, Sumanta Das

**Affiliations:** Civil and Environmental Engineering, University of Rhode Island, Kingston, RI 02881, USA; glyngdoh@uri.edu (G.A.L.); samidoner34@uri.edu (S.D.)

**Keywords:** wollastonite microfibers, cementitious composites, dynamic compressive behavior, finite element analysis, continuum micromechanics

## Abstract

This paper investigates the dynamic compressive behavior of wollastonite fiber-reinforced cementitious mortars using multiscale numerical simulations. The rate dependent behavior of the multiphase heterogeneous systems is captured in a multiscale framework that implements continuum damage towards effective property prediction. The influence of wollastonite fiber content (% by mass) as cement replacement on the dynamic compressive strength and energy absorption capacity is thereafter elucidated. An average compressive strength gain of 40% is obtained for mortars with 10% wollastonite fiber content as cement replacement, as compared to the control mortar at a strain rate of 200/s. The rate dependent constitutive responses enable the computation of energy absorption, which serves as a comparative measure for elucidating the material resistance to impact loads. Approximately a 45% increase in the dynamic energy absorption capacity is observed for the mixture containing 10% wollastonite fibers, as compared to the control case. Overall, the study establishes wollastonite fibers as a sustainable and dynamic performance-enhanced alternative for partial cement replacement. Moreover, the multiscale numerical simulation approach for performance prediction can provide an efficient means for the materials designers and engineers to optimize the size and dosage of wollastonite fibers for desired mechanical performance under dynamic loading conditions.

## 1. Introduction

Portland cement concrete is one of the most widely used construction materials around the world. However, concrete performs poorly in terms of fracture response [1,2], and it shows an almost negligible tensile response [3,4]. The shortcomings are even more magnified under dynamic loading conditions [5,6]. Reinforcing ordinary Portland cement (OPC)-based systems with fibers exhibit enhanced tensile and flexural strengths with higher toughness [7,8,9,10]. These reinforcements are able to provide enhanced resistance against the initiation and propagation of micro and macro cracks [11]. Such enhancements in fracture response have been reported, involving the incorporation of various fibers in cementitious composites, including steel fibers [12,13], basalt fibers [14,15], polyvinyl alcohol fibers [16,17], waste carpet fibers [18,19], nano calcium carbonate [20,21], multiwalled nanotubes of carbon [22,23], two dimensional graphene sheets [24,25], etc. Strength enhancement has also been reported for the steel fibers embedded in concrete under compressive and tensile loading conditions [26,27,28]. Owing to the focus of the majority of such studies being either static or low-rate loadings, it is worthwhile to explore their high strain rate performance [10,29,30]. Such dynamic conditions can range from seismic events and offshore loadings to explosions [31,32]. The incorporation of steel fibers in concrete has shown to be beneficial in terms of strength enhancement under dynamic loading conditions [7,8,9,10,32]. Enhancements in dynamic compressive response are also observed in concretes with polypropylene reinforcement [33], CF (carbon fiber) reinforcement [34], and ceramic reinforcement [10]. While previous studies have reported dynamic strength enhancement and stiffness gains in concrete with fiber incorporation, these fibers are expensive, which limits their practical applicability in large-scale projects [35]. An alternative approach can be adopted using economical fiber alternatives, such as waste iron powder [32], recycled plastics [36,37], other recycled industrial products [36,38,39], or affordable natural fibers [40,41,42,43,44]. Natural fibers such as wollastonite, basalt, and wood have been shown to improve the mechanical performance of concrete under either quasi-static or low strain rates [41,42,45,46,47]. Wollastonite has gained much attention in the last decade due to its biocompatibility, low thermal expansion, low thermal conductivity, and thermal stability [48,49,50]. Wollastonite can also be artificially synthesized using SiO_2_ materials enriched with calcium [49,50,51,52]. While the above-mentioned previous studies have shown significant improvements in the mechanical performance of cementitious composites under quasi-static conditions [41,53], studies quantifying the compressive behavior of wollastonite reinforcement in cement-based compositions under dynamic loading conditions are still limited. This motivates the current study on evaluating the influence of wollastonite fibers in cementitious mortars under dynamic loading conditions.

The current study elucidates the compressive behavior of such systems under dynamic loads by carrying out simulations in a multiscale, finite element-based framework. The efficiency of wollastonite fiber incorporation is studied under dynamic loading conditions and quantified as peak compressive stress and energy absorbed until failure. Towards that end, dynamic compression is applied, and the constitutive response is evaluated by employing numerical homogenization and a strain rate dependent damage model [54,55] in a continuum framework that implements micromechanical simulations. While the matrix undergoes continuum damage, the inclusion–matrix interfaces adopt cohesive damage. The interfacial and matrix damages govern the pre-peak and post-peak behavior. The homogenized rate dependent behavior characterizes the effective constitutive response. In addition to performance enhancement, the wollastonite fibers partially replace OPC, which contributes to about 5–7% of global greenhouse gas emissions in the world [56]. Thus, in addition to the potential performance enhancement, the incorporation of wollastonite fibers as a partial replacement of OPC can also improve the sustainability credentials of concrete. Overall, this paper forwards viable avenues to develop wollastonite fiber-reinforced cementitious composites as a more sustainable and dynamic performance-enhanced alternative to traditional cementitious composites. Moreover, the developed numerical approach can be used as a starting point to elucidate various innovative avenues to tune the dosage, shape, and aspect ratio of these wollastonite fiber-reinforced cementitious composites for their desired performance.

## 2. Multiscale Numerical Simulations for Dynamic Response Prediction of Fiber-Reinforced Mortars

The rate dependent constitutive responses of wollastonite fiber incorporated cementitious systems are elucidated in this section using a continuum micromechanics-based numerical framework. The inherent heterogeneity in such systems is captured at multiple interactive length scales. For every scale, a representative geometry is generated to capture the heterogeneity at that scale. A homogenization procedure is adopted to elucidate the constitutive behavior under strain rates of the representative unit cells. The constitutive response thus ascertained serves as an input to the subsequent length scale. In the current scope of the study, the interactive length scales correspond to that of the fiber-reinforced cementitious systems, which are homogenized as a matrix with dispersed sand at the mortar scale, as elaborated in Supplementary Materials (Section A). The rate dependent response for the fiber incorporated mortars is thereafter correlated with the experimental observations. In the following subsections, the strategy for the framework is elaborated. This is followed by the rate dependent homogenized responses. The experimental data for some compositions are thereafter correlated. The analysis is carried out in ABAQUS^TM^ using python scripts for geometry generation and user-defined subroutines for material property assignment, followed by a post-processing module coded in MATLAB.

### 2.1. Multiscale Simulation Strategy

**Figure 1:**
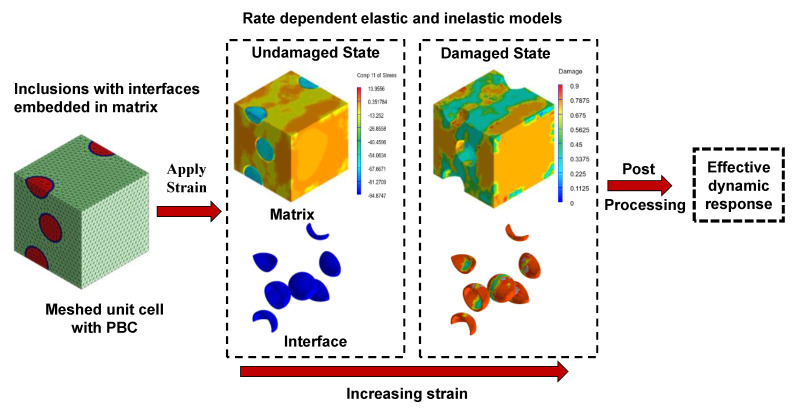
Schematic representation of the homogenization strategy.

The current section presents the simulation strategy towards a comparative dynamic performance evaluation of wollastonite fiber incorporated mortars. The first step involves capturing the material heterogeneity with representative unit cells that capture the geometrical attributes of the microstructure. The unit cells implement periodic boundary conditions (PBCs). A multiphase meshing strategy is adopted thereafter. A strain along the axis of the unit cell is, then, applied at a finite rate, and the stress response is recorded. A strain rate dependent damage governs the post-peak behavior of the unit cell in the strategy. Thus, the constitutive response is acquired at every respective length scale. The scheme is shown in Figure 1, which elaborates the homogenization approach. The following subsections further elaborate on the process for a comprehensive understanding.

#### 2.1.1. Representative Geometry Generation

In this study, the unit cell for the respective length scale is generated using the hard particle contact model, also called Lubachhevsky–Stillinger algorithm [57,58]. The computer program ensures no overlap between particles. The program has been carried out and adequately detailed in the authors’ previous publications [32,59,60,61,62,63]. Additional details are provided in the Supplementary Materials (Section B).

#### 2.1.2. Periodically Bounded Unit Cells

After meshing the representative geometry using a python script, periodic boundary conditions (PBCs) [60,62,64] are implemented. Under such boundary conditions, both the displacements and tractions are continuous in neighboring cells. The continuity is ensured across the boundaries of such cells. Such BCs are implemented in the previous studies for random heterogeneous systems using FE analyses [32,61,62,63,65]. Owing to the efficiency PBCs offer during computation, smaller unit cells with faster convergence can be achieved [60]. Detailed analysis of PBCs can be found in [60,61,66]. For ease of reference, more details on PBCs are provided in Section C of the Supplementary Materials.

#### 2.1.3. Constitutive Behavior Prediction

The meshed RVEs are subjected to compressive strain at a strain rate of 200/s. This enables the framework to emulate displacement control during experimental loading. The response of the homogenized composite at the pre-peak regime is dominantly characterized by the dynamic modulus of the matrix, which is a function of strain rate expressed as [54]

(1) $$E=\left[C_{1}+C_{2}{\left(\frac{\dot{\varepsilon_{d}}}{\dot{\varepsilon_{s}}}\right)}^{C_{3}}\right]$$

where $C_{1}$, $C_{2}$, and $C_{3}$ are material parameters, $\dot{\varepsilon_{d}}$ is the strain rate for dynamic loads, and $\dot{\varepsilon_{s}}\;\left(=3\times 10^{-6}\right)$ is the quasi-static strain rate.

#### 2.1.4. Rate Dependent Damage

The elastic constitutive behavior is determined by the dynamic modulus, whereas strain rate governed damage determines the inelastic response. In this study, a rate dependent damage model in [54,55] is adopted. For isotropic damage, the constitutive equation incorporating the damage variable can be expressed as [54,55]

(2) $$\sigma_{d}\left(\dot{\varepsilon },\varepsilon \right)=\left[1-D\right]\sigma \left(\dot{\varepsilon },\varepsilon \right)$$

where $\sigma_{d}$ and σ correspond to damage stress and undamaged stress as a function of strain (ε) and strain rate ($\dot{\varepsilon }$), respectively. A damage variable D, which is a function of ($\dot{\varepsilon },\varepsilon$), takes a value between 0 and 1, where zero means no occurrence of damage and one corresponds to a material when the crack is fully propagated. The damage evolution (see Equation (3)) follows a non-linear relationship with the strain rate.

(3) $$\dot{D}=C_{4}{\dot{\varepsilon }}^{\lambda }+C_{5}\dot{\varepsilon }$$

For a constant strain rate, the damage parameter D is obtained by integrating Equation (3) with respect to time, which can be written as

(4) $$D=(C_{4}{\dot{\varepsilon }}^{\xi }+C_{5})\varepsilon +C_{6};\;\xi =\lambda -1$$

Using an initial boundary condition where $D{|}_{\varepsilon =0}=0$, Equation (4) yields $C_{6}=0$. Thus, D can be further expressed as

(5) $$D=(C_{4}{\dot{\varepsilon }}^{\xi }+C_{5})\varepsilon ;\;\varepsilon >\varepsilon_{D_{0}}$$

The values $C_{4}$, $C_{5}$, and ξ are material constants. Thus, beyond $\varepsilon_{D_{0}}$, the damage is characterized as given in Equation (5). It is to be noted that $\varepsilon_{D_{0}}$ is the strain at which damage is initiated. A user-defined subroutine implements the aforementioned damage model in ABAQUS^TM^.

#### 2.1.5. Post-Processing

A post-processor coded in MATLAB computes the volume-averaged stresses in the unit cells corresponding to every strain state. An iterative process is implemented to compute the homogenized constitutive response for each strain rate (quasi-static and dynamic). Since the stiffness degrades with damage evolution, both pre-peak and post-peak responses can be similarly obtained. The subsequent section implements the simulation strategy explained herewith. The material composition for the demonstration includes wollastonite micro-reinforcements in cement-based systems. Under dynamic loading conditions, the effective constitutive behavior aims to draw a contrast with control composition, thus explicating the benefits of wollastonite incorporation.

## 3. Results and Discussion

### 3.1. Representative Length Scales for Numerical Simulation

**Figure 2:**
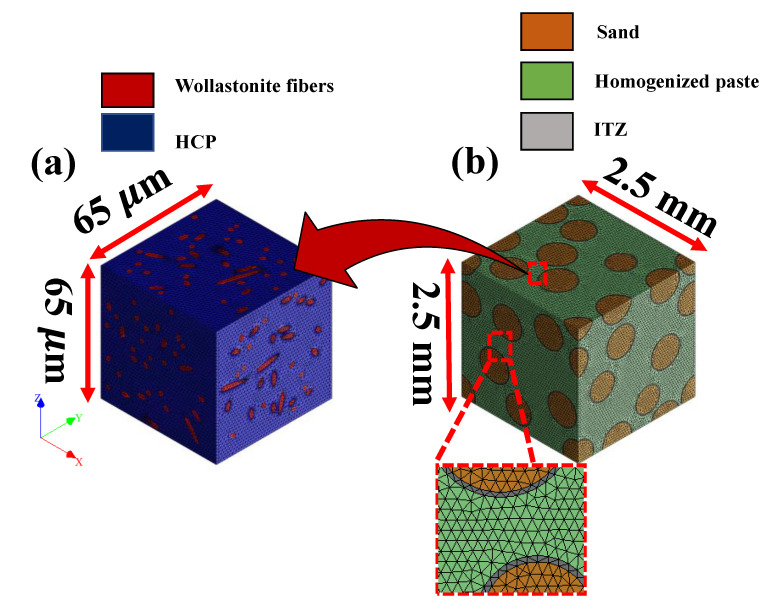
Representative geometry at (**a**) paste scale with 10% wollastonite fibers in HCP matrix and (**b**) mortar scale with sand inclusions in homogenized paste.

The two scales in the study correspond to that of the paste scale and, subsequently, the mortar scale, as shown in Figure 2. Figure 2 shows the two length scales for a representative mortar with a 10% wollastonite fiber. The wollastonite fibers have an average size of 4 µm [41]. The average length of the fibers is 12 µm with an aspect ratio of three. For sand inclusions, the median size of the sand is 600 µm, adopted after [32]. The homogenization algorithm is applied both at the cement paste scale and the mortar scale to ascertain the homogenized stress–strain response. In the cement paste scale (Figure 2a), the fibers are, first, homogenized with the hardened cement paste (HCP) matrix. The effective behavior thus obtained corresponds to wollastonite-reinforced pastes. The choice of unit cell size and the mesh convergence are provided in the Supplementary Materials, Section A.

The homogenized properties from the paste scale are assigned in the mesoscale (refer to Figure 2b) as input property of the matrix in which sand inclusions are embedded. The responses obtained at the mortar scale represent the effective strain rate dependent compressive constitutive response of fiber incorporated mortars. While no interfacial areas between the fiber and HCP are considered due to lack of data [67], the interfacial transition zones (ITZ) at the sand–matrix interfaces are successfully implemented herein. The adopted thickness of ITZ is 20 µm, which is also experimentally observed in the cementitious mortar scale [68,69]. The fiber and sand inclusions are modeled as linear elastic due to their significantly higher strength than that of the matrix. Such an approach has also been adopted in various previous studies. The mesh convergence studies can be found in the Supplementary Materials (Section A). The strength is computed from the constitutive response as the peak stress, while the area under the stress–strain curve is computed as the absorbed energy. The DIF is computed as the ratio of the strengths under dynamic and quasi-static loads. The multiscale homogenization is implemented at both the paste and mortar scales in the forthcoming subsections that characterize the behavior of the wollastonite incorporated mortars.

### 3.2. Quasi-Static Compressive Behavior

The quasi-static compressive strength of the control and wollastonite microfiber-reinforced mortars are determined using a constant strain rate of 1 × 10^−5^/s. The material parameters used for hardened cement paste (HCP) are presented in Table 1. For a comparative performance evaluation, the quasi-static modulus of wollastonite fibers is considered 300 GPa [41] while that of sand is 70 GPa [32]. The inclusions and the matrix have a Poisson’s ratio of 0.2.

**Figure 3:**
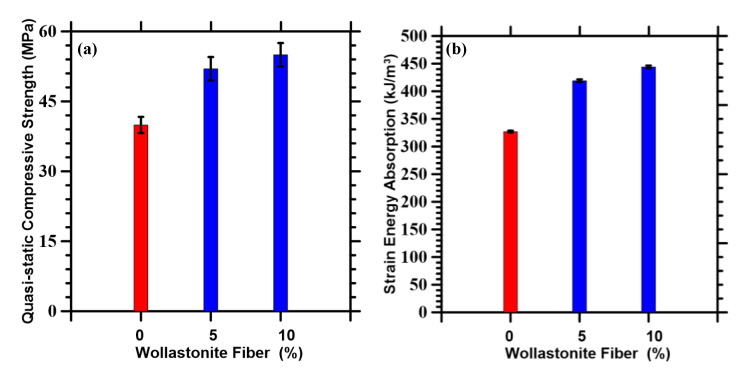
(**a**) Quasi-static compressive strengths and (**b**) strain energy absorption of control (shown in red) and wollastonite fiber-reinforced mortars (shown in blue).

As explained earlier, the homogenized wollastonite fibers and HCP matrix is used as the input property of the matrix for the mortar scale to obtain the effective compressive strength under quasi-static conditions for the mortars. Figure 3a shows the compressive strength of control and fiber-reinforced mortars for quasi-static loads. For the fiber-reinforced mortars, a consistent compressive strength gain of about 35% is observed with respect to the control specimen. As the cement is replaced with the wollastonite fibers, the strength gain due to the fibers roughly compensates for the loss due to cement replacement thus producing similar values of strength with increasing fiber content. The wollastonite microfibers are also likely to act as fillers [55] that contribute to strength enhancements [41]. In this paper, the wollastonite fiber content was not increased beyond 10%, since such mixtures did not provide adequate rheological behavior, as noted elsewhere [41]. The specific energy absorption (see Figure 3b) is defined as the integral of the stress response with strain. It is observed that the energy absorption capacity increases with increasing fiber content, as contrasted with the control mortar.

It is worth mentioning that the simulated quasi-static compressive strength for the control and wollastonite-reinforced specimens obtained in this study lies in the experimental ranges of 45 ± 5 MPa and 55 ± 5 MPa, respectively [41,71]. The good correlation, as observed, serves to enhance the confidence in the numerical simulation presented herewith.

### 3.3. Compressive Response under Dynamic Loads

Having ascertained the quasi-static responses of the wollastonite fiber incorporated systems where the fibers reinforce the otherwise brittle cementitious matrix, this section evaluates the trends for dynamic loading conditions. Besides obtaining the constitutive behavior under a high rate, the dynamic increase factors are computed that embody the high-rate deformation characteristics.

**Figure 4:**
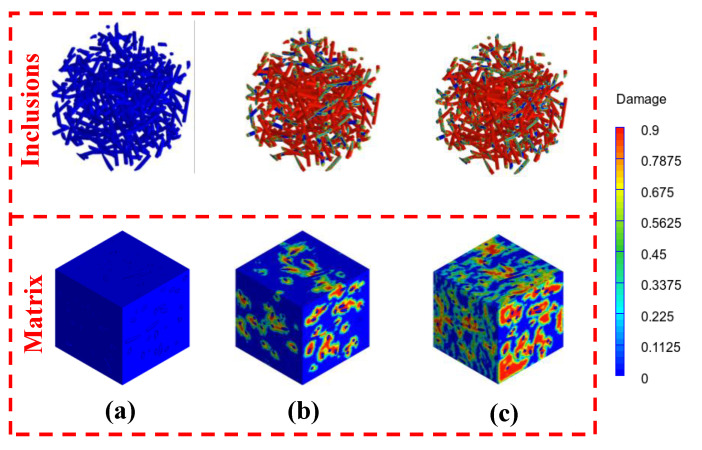
Damage states at 200/s rate with increasing strain for the mortar with 10% fiber content at strain states of (**a**) undeformed, (**b**) 0.0027, and (**c**) 0.0045.

The homogenized numerical results obtained from multiscale analyses are demonstrated in this section. In this study, the strain is applied along the x-direction, and the time steps are implemented to simulate a 200/s loading rate, as applied on a periodically meshed unit cell (refer to Figure 4a). The parameters characterizing the material inputs for control HCP as a function of strain rate are reported in Table 1. To this end, these parameters are used for the HCP matrix at a paste scale when simulated with wollastonite fibers. The Young’s modulus of wollastonite fiber from a quasi-static experiment lies between 300–530 GPa [41]. For a comparative performance evaluation, the quasi-static modulus is considered 300 GPa. Here, a gain of 53% in Young’s modulus is considered [72], as compared to a quasi-static modulus of wollastonite fiber for the stain rate of 200/s. The phases adopt a Poisson’s ratio of 0.2. Studies have shown insignificant changes for such when the Poisson’s ratio is varied in between 0.18–0.22 [65,73]. The progressive damage, as implemented in ABAQUS™, is illustrated in Figure 4 for a strain rate of 200/s.

Figure 4a–c show the progressive damage states observed as the strain is increased from zero to 0.0027 and 0.0045. The damage onset and evolution are depicted in Figure 4b,c, respectively. Table 2 reports the material parameters for wollastonite-reinforced pastes. These material parameters at the microscale are implemented to define the material model for the matrix in the mortar scale.

The dynamic modulus of the sand–matrix interface (ITZ) elements is considered as 40% of that of the matrix [68,74]. As the cracking strain for ITZ is unknown, an inverse analysis is performed. In such an analysis, the volume fraction of sand is 50%. The three phase unit cells have sand inclusions surrounded by a 20 µm thick ITZ. The inverse analysis results in a cracking strain $\varepsilon_{D_{0}}^{ITZ}$ of 0.00048 for ITZ. The cracking strain corresponds to an applied strain of $\varepsilon_{D_{0}}^{M}/4$, which is reported as the strain state for initiation of debonding [70]. The matrix cracking strain, denoted by $\varepsilon_{D_{0}}^{M}$, equals 0.001 for HCP [70]. The damage in ITZ evolves in a rate dependent fashion, as explained earlier. The material parameters of the ITZ besides the dynamic modulus and the $\varepsilon_{D_{0}}^{ITZ}$ are considered equal to that of cement paste, owing to limited data available in the literature. For sand inclusions, the secant modulus follows a linear relation with the logarithmic strain rate [75], with the modulus under quasi-static loads at 70 GPa [32]. The rate dependent modulus is obtained by a gain of 50% for a strain rate of 200/s [32].

**Figure 5:**
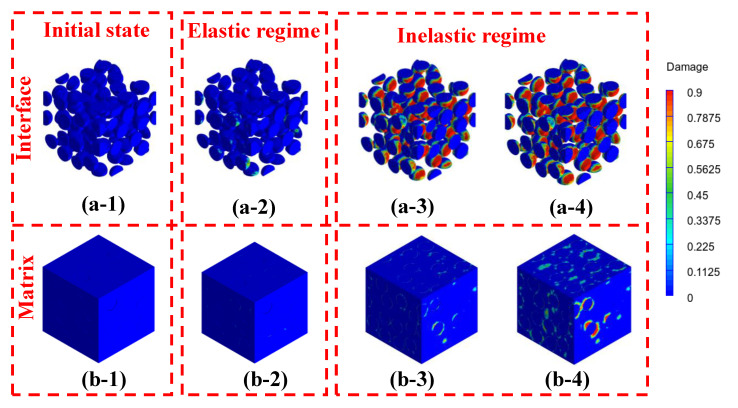
Progressive damage for mortar composition with 10% wollastonite fibers for a strain rate of 200/s at (**a**) interface and (**b**) matrix in fiber; the strain states correspond to (**a-1**) and (**b-1**) undeformed, (**a-2**) and (**b-2**) at 0.0018, (**a-3**) and (**b-3**) at 0.0033, and (**a-4**) and (**b-4**) at 0.0044.

Figure 5 presents the progressive damage states at the mortar scale for the mortar mixture of 10% at a strain rate of 200/s. While Figure 5(a-1–a-4) present the progressive interfacial damage, Figure 5(b-1–b-4) depict the corresponding progressive damage in the matrix. Figure 5(a-1,b-1) correspond to the undeformed state. Figure 5(a-2,b-2) correspond to a strain of 0.0018, which is under elastic regime from the global response. Figure 5(a-3,b-3) correspond to the interfacial and matrix damages at a strain of 0.0033. Similarly, Figure 5(a-4,b-4) correspond to the progressive interfacial and matrix damage at a strain of 0.0044. It is observed that the interfacial damage initiates much earlier before reaching the peak global strain. The interfacial damage continues to propagate as the applied strain increases. This process stops when the stress in the matrix exceeds its compressive strength, beyond which the matrix damage initiates and starts propagating, defining the post-peak regime.

**Figure 6:**
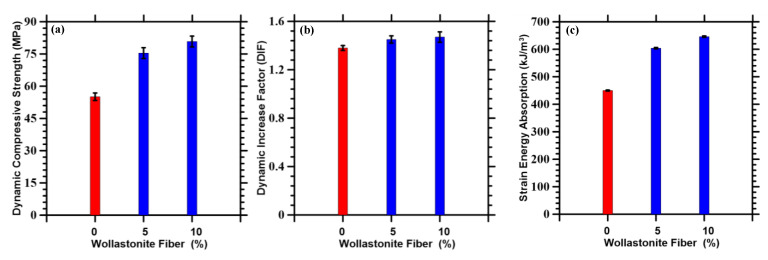
Dynamic properties: (**a**) compressive strength, (**b**) dynamic increase factor, and (**c**) strain energy absorption for varying wollastonite content at a strain rate of 200/s.

Figure 6 shows the comparative performance of the wollastonite fiber incorporated cementitious systems at a strain rate of 200/s. The trends observed for dynamic loading cases are consistent with the ones obtained for quasi-static loadings. The mixtures with wollastonite fibers show higher peak loads than does the control mixture. Figure 6a summarizes the dynamic compressive strengths of the wollastonite fiber incorporated mortars, as compared to the control mortar. While the fiber incorporated systems show an average strength gain of 40%, the trends are consistent with those observed for quasi-static loadings. In order to understand the rate effect, it is worthwhile to compute the dynamic increase factors, which is the ratio of the strengths at a high strain rate to the quasi-static strengths, expressed as a ratio. For every composition, the rate effects can be exemplified by their respective dynamic increase factors. The fiber incorporated specimens show significantly higher dynamic increase factors, as compared to the control specimens (see Figure 6b), thus, substantiating their applicability for superior dynamic performance. It is observed in Figure 6c that the fiber-reinforced mixtures show significantly higher energy absorption, as compared to the control case. Such an enhancement in energy absorption can be attributed to crack bridging and/or deflection effects of wollastonite fibers.

While the experimental dynamic increase factor for compressive strength in mortar reported in the literature is 1.57 ± 0.08 [71], the simulated dynamic increase factor obtained is 1.54. Thus, an excellent correlation is observed with the current numerical simulation framework.

## 4. Conclusions

The multiscale numerical simulation methodology presented in this paper enables the development of an efficient predictive tool toward the design of these wollastonite microfiber-reinforced cementitious systems for efficient dynamic performance. In order to capture the hierarchical geometrical features at multiple length scales, numerical homogenization is employed at each scale. A rate dependent damage evolution in a continuum mechanics framework is implemented on periodically bound unit cells. This facilitates computation of effective dynamic behavior of the fiber-reinforced heterogeneous systems. While the numerical simulation results clearly demonstrate a significant enhancement in compressive strength and energy absorption capacity under dynamic loading conditions for wollastonite-reinforced mortars, it needs to be noted that such trends should be confirmed by experimental studies. Nevertheless, in the absence of experimental results, the numerical results presented in this paper can be used as a starting point for the design and development of such wollastonite fiber-reinforced cementitious composites. Such a performance enhancement under dynamic loading conditions can be attributed to the presence of wollastonite microfibers that act as micro-reinforcements to bridge the microcracks in the matrix. Additionally, the wollastonite microfibers act as fillers that block the voids, resulting in strength enhancement. Such performance enhancement can pave the way for the utilization of wollastonite as a viable fiber alternative under high strain rate conditions. In addition, the numerical approach presented here can be adopted as a starting point for a microstructure-optimized design of wollastonite fiber incorporated cementitious composites and their upscaling to concretes for enhanced performance under dynamic loading conditions.

## Figures and Tables

**Table 1:** Material parameters for HCP [54,70].

$C_{1}$	$C_{2}$	$C_{3}$	ξ	$C_{4}$	$C_{5}$	$\varepsilon_{D_{0}}$
5064.1	4.71	1.70	0.487	0.019	0.657	0.001

**Table 2:** Homogenized material properties for wollastonite fibers in HCP.

Fiber Content (%)	$C_{1}$	$C_{2}$	$C_{3}$	ξ	$C_{4}$	$C_{5}$	$\varepsilon_{D_{0}}$
5%	6501	3.92	1.691	0.468	0.014	0.687	0.0014
10%	10,298	4.53	1.701	0.502	0.015	0.702	0.0018

## Data Availability

Data is contained within the article or Supplementary Material.

## Supplementary Materials

The following are available online at https://www.mdpi.com/article/10.3390/ma14164435/s1. Figure S1: (a) Results of the mesh-convergence study; (b) RVE size study (edge length of RVE) for predicted Young’s modulus of 5% wollastonite fiber replacing cement at paste scale. Figure S2. (a) Mesh convergence study and (b) RVE size study (Edge length of RVE) for predicted Young’s modulus along three orthogonal directions for mortar.
