# Supplementary material for: Finite Element-Based Numerical Simulations to Evaluate the Influence of Wollastonite Microfibers on the Dynamic Compressive Behavior of Cementitious Composites
Source: Materials (Basel). 2021 Aug 8;14(16):4435. doi: 10.3390/ma14164435 (PMC8399284; doi:10.3390/ma14164435)
Supplement: Supplementary file 1 [file materials-14-04435-s001.zip › materials-1315136-supplementary.pdf]

materials

Supporting information

Finite Element-Based Numerical Simulations to Evaluate the Influence of Wollastonite Microfibers on the Dynamic Compressive Behavior of Cementitious Composites

Gideon A. Lyngdoh, Sami Doner, Sumeru Nayak * and Sumanta Das *

Civil and Environmental Engineering, University of Rhode Island, Kingston, RI 02881, USA; glyngdoh@uri.edu (G.A.L.); samidoner34@uri.edu (S.D.)
* Correspondence: sumeru@uri.edu (S.N.); sumanta_das@uri.edu (S.D.)

A. RVE Size and Mesh Convergence Study

A.1 Wollastonite Fiber Reinforced Cement Paste Scale

In order to generate representative unit cells for the wollastonite fiber reinforced cement pastes, mesh-convergence and RVE size studies are carried out. Figure S1a shows the results of the mesh-convergence study.

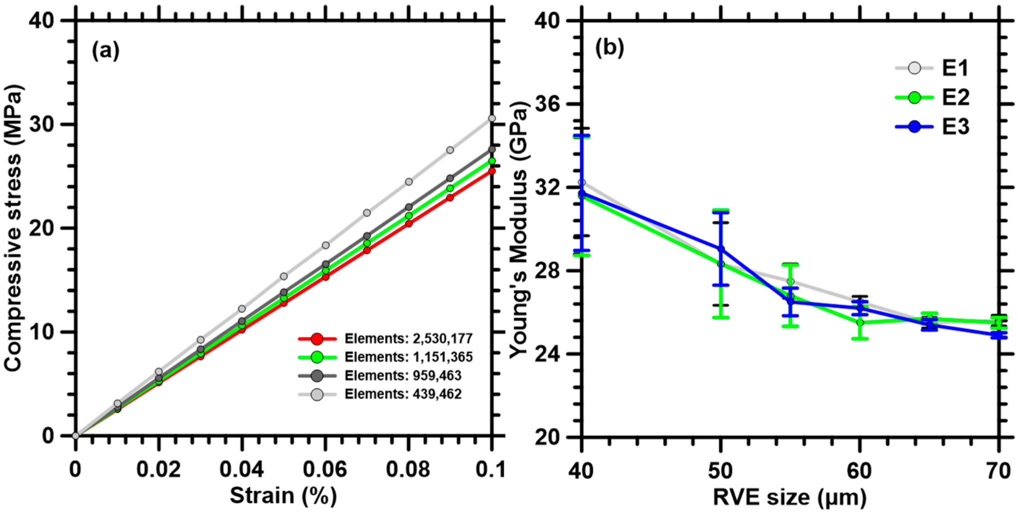

Figure S1. (**a**) Results of the mesh-convergence study; (**b**) RVE size study (edge length of RVE) for predicted Young's modulus of 5% wollastonite fiber replacing cement at paste scale.

A mesh with 2,530,177 ten-node tetrahedral elements (C3D10 in ABAQUS), yields a converged solution. Thus, a total of 1,151,365 elements are implemented. Figure S1b shows the homogenized Young's modulus with a varying edge length of RVEs. Overall, an RVE edge length size of 65 μm yields converged solutions. Thus, the chosen RVE size is 65 μm for adequately capturing the isotropic behavior of the wollastonite-paste unit cell. The chosen size invokes a trade-off between computational expense and prediction efficiency.

A.2 Wollastonite Fiber Reinforced Mortar Scale

To ensure the representativeness of the unit cells and the adequacy of the mesh at the mortar scale, mesh-convergence and RVE-size sensitivity studies are carried out as shown in Figure S2.

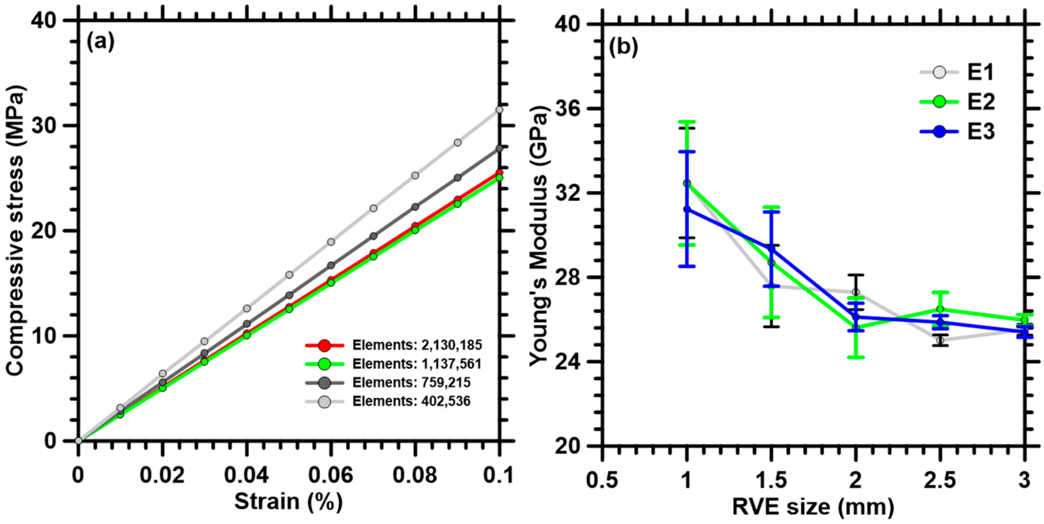

Figure S2. (**a**) Mesh convergence study and (**b**) RVE size study (Edge length of RVE) for predicted Young's modulus along three orthogonal directions for mortar.

Figure S2a shows the results of the mesh-convergence study and a mesh containing 1,137,561 ten-node tetrahedral elements (C3D10 in ABAQUS) yields converged solution. Towards establishing the adequate RVE size, ten RVEs are generated with varying every edge length. Thereafter, uniaxial tests are carried to calculate the homogenized Young's modulus for every size of unit cell. Figure S2b shows the variation of the predicted responses with varying RVE size. It can be observed that the fluctuations, represented by error bars, are higher for smaller RVEs which eventually die out with a sufficiently large RVE size. Additionally, the directional properties converge for RVE sizes ≥2 mm. Thus, the chosen RVE size of 2.5 mm is adequate to capture the isotropic behavior of the sand-HCP unit cell. The chosen size is adopted based on a trade-off between computational expense and prediction efficiency.

B. Unit Cell Generation Approach

For generation of representative unit cells, the point inclusions are first randomly dispersed inside the periodic bounding box with some initial velocities. The radius of each particle (r_i) is considered as a function of the growth rate (g_i) and is initialized as zero. This growth rate is tailored to attain the desired particle size distribution. The growth rate is expressed as:

$$\frac{dr_i}{dt} = g_i \tag{1}$$

where $i = 1,2,3 \ldots n$(number of particles). The growth rate between the time interval t^n and t^{n+1} is solved using finite difference scheme as follows:

$$g_i = \frac{(r_i^n - r_i^{n+1})}{\Delta t} \tag{2}$$

where r_i^n and r_i^{n+1} are the radius of i^{th}particle at time interval t^n and t^{n+1}, respectively and $\Delta t = t^{n+1} - t^n$. The particle radii at t^{n+1} is updated by employing the growth rate and time increment (Δt) as follows:

$$r_i^{n+1} = r_i^n + g_i \Delta t \tag{3}$$

and the position of the ith particle at time t^{n+1} is updated with a constant velocity (v_i^n) between time nodes as:

$$\mathbf{x}_i^{n+1} = \mathbf{x}_i^n + \mathbf{v}_i^n \Delta t \tag{4}$$

The length vector (l_{ij}^{n+1}) that connects between the centers of particles 'i' and 'j':

$$\mathbf{l}_{ij}^{n+1} = \mathbf{x}_j^{n+1} - \mathbf{x}_i^{n+1} \tag{5}$$

The particles 'i' and 'j' are expected to overlap if the magnitude of length vector ($\|\mathbf{l}_{ij}^{n+1}\|$) is less than the sum of their radii. The timestep size is calculated as [1]

$$\Delta t = \min\left[\frac{-v \pm \sqrt{v^2 - uw}}{u}\right] \tag{6}$$

$$v = \mathbf{l}_{ij}^{n}.\left(\mathbf{v}_j^n - \mathbf{v}_i^n\right) - (r_j^n + r_i^n)(g_i + g_j) \tag{7}$$

$$u = \left(\mathbf{v}_j^n - \mathbf{v}_i^n\right)^2 - (g_i + g_j)^2 \tag{8}$$

$$w = \mathbf{l}_{ij}^{n}.l_{ij}^{n} - (r_j^n + r_i^n)^2 \tag{9}$$

Here, $\mathbf{v}_j^n$ and $\mathbf{v}_i^n$ are the velocities of particles 'i' and 'j' at time t^n. $\mathbf{r}_j^n$ and r_i^n are the radii of particles 'i' and 'j' at time t^n. g_i and g_j are the growth rates for particles 'i' and 'j' at time t^n. l_{ij}^n is the length vector between the two particles 'i' and 'j' at time t^n. The timestep obtained from Equation (6) is performed for each particle pair that are being able to collide. The minimum timestep for all the possible collision is adopted to move forward for the next event. The new position x_j^{n+1} for particle 'i' is then updated using the forward Euler scheme as described in Equation (4) and new search for the next collision is started and likewise for other particles. The post-contact velocities are computed as:

$$v_{ni}^{n+1^+} = \min(v_{ni}^{n+1^-}, v_{nj}^{n+1^-}) - g_i \tag{10}$$

$$v_{nj}^{n+1^+} = \min(v_{ni}^{n+1^-}, v_{nj}^{n+1^-}) + g_i \tag{11}$$

Where $v_{ni}^{n+1^+}$ and $v_{ni}^{n+1^-}$is the velocity of the particle 'i' after the contact and before the contact, respectively. The normal contact velocity is expressed as:

$$v_n^{n+1} = \mathbf{v}^{n+1}.\mathbf{n}_{ij}^{n+1} \tag{12}$$

$$\mathbf{n}_{ij}^{n+1} = \mathbf{l}_{ij}^{n+1} \Big/ \|\mathbf{l}_{ij}^{n+1}\| \tag{13}$$

Thus, all the steps are repeated, and the particles change position in the bounding box, collide and grow to obtain desired volume fraction during the iteration process. The algorithm is an iterative process and terminated as soon as the target volume fraction is reached.

C. Periodic Boundary Conditions

Periodic boundary conditions are applied to implement the repetitive nature of the unit cells at each scale. The efficiency of such boundary conditions towards effective property prediction of inclusion embedded systems with various planes of symmetry are detailed in [2]. The relative displacement (x_k for $k = 1,2,3$) for each pair of nodes on the parallel boundary surfaces (represented by point A and point B lying on such faces) of the unit cell are given by the following Equation (4).

$$u_k^A - u_k^B = \tilde{\varepsilon}_{avg}\left(x_k^A - x_k^B\right) = \tilde{\varepsilon}_{avg} L_{RVE} \tag{14}$$

Where $\tilde{\varepsilon}_{avg}$ is the average strain in the unit cell, x is the position vector of the points A and B on the parallel boundaries, and L_{RVE} is the edge length of RVE. Equation (14) is

enforced in the simulation by setting constraint equations that relate the degree of freedom (DOF) of each pair of nodes on opposite boundaries to the 6 DOFs of a master node on parallel faces. The node pairs are correlated by a preprocessor to ensure the correspondence between such nodes on parallel surfaces. Such an implementation has been demonstrated to maintain traction and displacement continuity on the parallel surfaces [3]. A uniaxial compressive response can be elicited from the unit cells by assigning a displacement to one set of parallel faces. While the boundary conditions for the electrical simulations are periodic in Y and Z, a potential gradient is applied along X.

D. Wollastonite Microfiber Properties

The wollastonite fibers have an average size of 4 μm [4]. The average length of the fibers is 12 μm with an aspect ratio of 3. Table S1 shows the material properties.

Table S1. Properties of wollastonite microfiber inclusions.

Quasi-Static			Dynamic	
**Young's Modulus (GPa)**	**Poisson's Ratio**	**Gain**	**Young's Modulus (GPa)**	**Poisson's Ratio**
300	0.2	53%	459	0.2

References

1. Meier, H.A.; Kuhl, E.; Steinmann, P. A note on the generation of periodic granular microstructures based on grain size distributions, *Int. J. Numer. Anal. Methods Geomech*. **2008**, *32*, 509–522. https://doi.org/10.1002/nag.635.
2. Li, S. Boundary conditions for unit cells from periodic microstructures and their implications, *Compos. Sci. Technol.* **2008**, *68*, 1962–1974. https://doi.org/10.1016/j.compscitech.2007.03.035.
3. Xia, Z.; Zhou, C.; Yong, Q.; Wang, X. On selection of repeated unit cell model and application of unified periodic boundary conditions in micro-mechanical analysis of composites, *Int. J. Solids Struct*. **2006**, *43*, 266–278. https://doi.org/10.1016/j.ijsolstr.2005.03.055.
4. Dey, V.; Kachala, R.; Bonakdar, A.; Mobasher, B. Mechanical properties of micro and sub-micron wollastonite fibers in cementitious composites, *Constr. Build. Mater*. **2015**, *82*, 351–359.
